# Trust in the Internet as a Health Resource Among Older Adults: Analysis of Data from a Nationally Representative Survey

**DOI:** 10.2196/jmir.1552

**Published:** 2011-02-16

**Authors:** Donna M Zulman, Matthias Kirch, Kai Zheng, Lawrence C An

**Affiliations:** ^6^School of InformationUniversity of MichiganAnn Arbor, MIUnited States; ^5^Department of Health Management and PolicySchool of Public HealthUniversity of MichiganAnn Arbor, MIUnited States; ^4^Center for Health Communications ResearchUniversity of MichiganAnn Arbor, MIUnited States; ^3^Department of Internal MedicineUniversity of MichiganAnn Arbor, MIUnited States; ^2^Department of Veterans AffairsHealth Services Research and Development Center of ExcellenceAnn Arbor, MIUnited States; ^1^The Robert Wood Johnson Foundation Clinical Scholars ProgramUniversity of MichiganAnn Arbor, MIUnited States

**Keywords:** Older adults, Trust, Online health information

## Abstract

**Background:**

Distrust in the Internet as a source of health information remains common among older adults. The influence of this distrust on Internet use for health-related purposes, however, is unclear.

**Objective:**

The objective of our study was to explore how older adults’ trust in the Internet influences their online health-related activities, and to identify potential targets for improving health-related Internet resources for older adults.

**Methods:**

Data were obtained from a nationally representative, random digit-dial telephone survey of 1450 adults 50 years of age and older in the United States. A model was developed to conceptualize the hypothesized relationships among individual characteristics, distrust, and avoidance of the Internet as a health resource. Multivariate logistic regression analyses were conducted to examine the association between trust in online health information and use of the Internet for health-related purposes. Additional multivariate logistic regression analyses were conducted to identify the key characteristics associated with trust in online health information, adding sequentially the variables hypothesized to account for distrust among older adults: sociodemographic and health characteristics, inexperience and technical difficulties with the Internet, negative feelings toward the Internet, and lack of awareness about the sources providing the health information found online.

**Results:**

The mean (SD) age of the study population was 63.7 (10.6) years. Of the 823 (56.8%) Internet users, 628 (76.3%) reported using the Internet as a health resource. Trust in the Internet as a source of health information was associated with using the Internet for a number of health activities, including searching for information about a specific health condition (adjusted OR 4.43, *P* < .001), purchasing prescription drugs (adjusted OR 2.61, *P* = .03), and talking with a health care provider about information found online (adjusted OR 2.54, *P* = .002). Older adults (age ≥65 years) were less likely to trust the Internet as a source of health information (OR 0.63, *P* = .04), even after adjusting for other sociodemographic characteristics and health and function. This age effect was only slightly attenuated (adjusted OR 0.69, *P* = .13) after adjusting for inexperience and technical difficulties with the Internet, but it disappeared entirely (adjusted OR 0.96, *P* = .91) after adjusting for other hypothesized contributors to distrust (including finding the Internet confusing because it provides “too much information,” and lacking awareness about the source providing health information found online).

**Conclusions:**

Website design features that clearly identify the source and credibility of information and minimize confusion may build trust among older adults and offer an opportunity to increase the utility of the Internet as a health resource for this population.

## Introduction

The Internet is a potentially important source of health information, providing accessible resources on topics ranging from specific diseases and treatment options, to health care providers and insurance plans, to healthy lifestyle choices and health products. The number of adults in the United States who report using the Internet as a source of health information increased from 25% in 2000 to 61% (or 83% of Internet users) in 2008 [1]. In fact, the Internet has become the first source that many people turn to for information about certain health conditions [2].

Older adults, a population in which the vast majority have one or more chronic health conditions [3], could stand to benefit tremendously from the convenient and inexpensive health resources provided by the Internet. Nevertheless, this group remains a relative minority in their direct use of online health information [4-6], with a recent Pew Internet survey finding that only 27% of adults aged 65 years and older and 59% of adults aged 50 to 64 years look online for information about health and medical issues (compared to 71%-72% of adults aged 18-49 years) [1].

There are several possible explanations why use of Internet health resources remains low among older adults. First, barriers such as inexperience with technology or physical limitations may restrict computer usage by some individuals. For example, certain resources may be inaccessible due to website design factors such as small font size, overwhelming amounts of information, cluttered webpages, and lack of instructions [7,8]. Second, older individuals might prefer traditional sources of health information, such as physicians and pharmacists, over less familiar sources such as the Internet. These traditional sources of health information are sometimes referred to as “intermediaries,” experts who act as middlemen, providing consumers with the information that they seek. Online health resources, on the other hand, can be considered “apomediaries,” because they steer consumers to desired health information without standing between them [8]. While such resources have a number of benefits, they demand a certain level of knowledge, interest, and self-efficacy from users. Older adults, especially those with limited experience using technology, may have higher rates of computer-related anxiety and low computer self-efficacy, both of which correlate with slow technology adoption [9]. Further, these characteristics may lead to low levels of autonomy, resulting in a preference for more traditional sources of health information [8].

Finally, trust is likely to be another key factor in determining whether the Internet is a preferred source of health information [10]. While a universal definition of trust remains elusive among social scientists [11,12], it is generally accepted that the need for trust arises in the setting of risk, and that trust involves confidence in the reliability of an entity. Studies have shown that older adults are less likely than their younger counterparts to trust the Internet for health information [6,13,14]. One reason for this distrust may be due to difficulties assessing the credibility of online information sources. Credibility, or the believability, of a source is made up of two dimensions: trustworthiness (as subjectively perceived by the Internet user) and expertise (also subjectively perceived by the user, but sometimes influenced by objective characteristics such as comprehensiveness of information or sponsor’s credentials [15,16]). Older adults, who may be accustomed to trusting a health care provider for information, might find the process of assessing credibility of online material overwhelming, leading to general distrust in the Internet for any health-related purpose.

Distrust in online health information may be protective in certain circumstances [17], creating a motivating force for caution in a setting where not all sources are reliable. In 1997, an editorial in the *Journal of the American Medical Association* warned, “Let the reader and viewer beware,” because when it came to medical information on the Internet, “Those seeking to promote informed, intelligent discussion often sit byte by byte with those whose sole purpose is to advance a political point of view or make a fast buck” [18; page 1244]. While this warning still holds true today, the growing availability of patient portals and patient-driven online health communities [1] is gradually moving us toward a world in which the majority of our health care transactions and most available health information will be online. In this scenario, distrust and other existing obstacles to the use of Internet health resources could become substantial barriers to health care access and quality [10].

**Figure 1:**
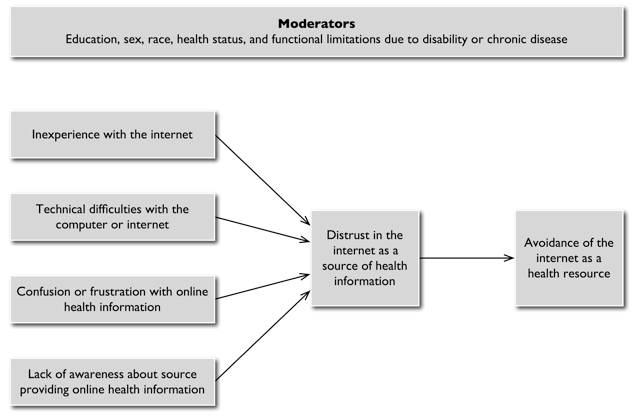
Characteristics hypothesized to influence distrust and avoidance of the Internet as a health resource among older adults

We sought to explore the role of trust in older adults’ use of the Internet as a health resource, using a nationally representative telephone survey that examined the utilization of and attitudes toward the Internet as a source of health information among adults 50 years of age and older. We developed a model that conceptualized potential relationships among individual characteristics, distrust in the Internet, and avoidance of the Internet as a health resource (Figure 1). We then performed a series of analyses in order to (1) explore the association between trust in the Internet and use of the Internet for health information and other health-related activities, and (2) identify potential targets for improving health-related Internet resources for older adults.

## Methods

Data for this study were obtained from a Kaiser Family Foundation survey of health-related Internet use among adults 50 years of age and older. Details of the survey have been published previously [19]. Briefly, the survey was designed in consultation with Princeton Survey Research Associates (PSRA). PSRA conducted the telephone interviews in English between March 5 and April 18, 2004. The sample was drawn using standard list-assisted random digit-dialing methodology. As many as 10 attempts were made to contact every sampled telephone number.

A nationally representative sample of 1450 adults aged 50 years and older were interviewed, including 583 respondents aged 65 years and older. The overall response rate was 38% (the contact rate was 82%, 51% of those contacted consented to an interview, and 90% of those who consented completed the interview). The interviewed sample was weighted to match national parameters established by the US Census Bureau’s 2003 Annual Social and Economic Supplement for sex, age, education, race, Hispanic origin, US region, and number of adults in the household age 50 years or older. The margin of sampling error for the complete set of weighted data was ±3%, and for those aged 65 years and older it was ±4%.

### Dependent Variables

#### Use of the Internet as a Health Resource

Survey respondents were asked a series of yes/no questions about whether they had ever used the Internet for a range of health activities, including obtaining information about a specific health condition or topic (including cancer, heart disease, arthritis, diabetes, Alzheimer’s disease, osteoporosis, high cholesterol, nutrition/exercise/weight loss, or mental health issues like depression or anxiety); obtaining information about a doctor, hospital, nursing home, home health agency, or other health care provider; looking for news about health policy issues; comparing prices for prescription drugs; and purchasing prescription drugs, vitamins, and supplements. Responses to these questions were analyzed first by constructing a single dichotomous dependent variable that captured any indication of having used the Internet as a health resource, and then in separate analyses in which each specific health-related activity was assessed as a dichotomous dependent variable. All respondents who indicated that they used the Internet for any health-related purpose were also asked whether online information had ever prompted them to change their behavior, to make a decision about a medical condition, to visit or talk to a health care provider, to change their health insurance plan, or to have a conversation with a friend or family member about the online health information.

#### Usefulness of the Internet as a Health Resource

Usefulness of the Internet as a health resource was assessed using the question, “How much has the information you have found on the Internet helped you take care of your health?” Responses were dichotomized as “somewhat” or “a lot” versus “only a little” or “not at all.”

#### Trust in the Internet as a Source of Health Information

All survey respondents were asked how much they trust the Internet “to provide accurate information about health problems or issues that are important to you.” Responses were dichotomized as “somewhat” or “a lot” versus “not too much” or “not at all.” The same question was also asked for other information sources, including health care providers, pharmacists, newspapers, magazines, books, television, radio, and friends or family. A mean trust score for non-Internet sources was calculated, and this score was used to adjust analyses for general trust in health information resources.

### Independent Variables

#### Sociodemographics

Sociodemographic characteristics included age (analyzed as a continuous variable, and dichotomized using the prespecified cutoff of 65 years), sex, race (dichotomized as white and nonwhite), and education (categorized as high school or less, some post-high school education, and college graduate).

#### Health and Functional Status

Respondents were questioned about their overall health using a 5-point scale (collapsed as fair or poor, versus excellent, very good, or good). Their overall functional status was assessed with a single question: “Does any disability, handicap, or chronic disease keep you from participating fully in work, school, housework, or other activities?”

#### Internet Experience and Technical Difficulties

Respondents who reported using the Internet were asked how many years have passed since they first started going online (dichotomized as >5 years and ≤5 years). They were also asked how often they have technical problems with their computer or Internet access (categorized as often, sometimes, not too often, or never).

#### Reasons for Distrust Among Older Adults

Three additional survey questions were selected as measuring potential reasons for distrust among older adults. Respondents who used the Internet to look for health information were asked whether they would describe their experience as “frustrating because it’s hard to find what I’m looking for,” or “confusing because there’s too much information.” They were also asked how often they “look to see who provides the health and medical information” they find on the Internet (dichotomized as always, most of the time, or sometimes, versus hardly ever or never). This last question was used to test our hypothesis that individuals who were aware of the source providing online health information would be more likely to trust the information they obtained.

### Statistical Analysis

Descriptive statistics of survey respondents’ Internet use have been published previously [19]. We conducted a multivariate logistic regression of Internet users (n = 823) in order to identify how trust is associated with a person using the Internet as a health resource, and with that person finding the Internet useful as a health resource. We adjusted our models for individuals’ health status and functional limitations, for inexperience and technical difficulties with the Internet, and for sociodemographics including age, sex, race, and education. Because of the large number of missing values for income (411/1450, 28.3%) and the strong correlation between income and education (*r* = 0.49), income was not included in any of our multivariate models.

We conducted additional analyses to identify the relationship between trust and a number of specific health-related Internet activities, including use of the Internet to obtain information about a specific health condition or health provider, to look for health policy news, and to purchase prescription drugs or make a treatment decision. These analyses were adjusted for sociodemographic and health characteristics.

Finally, we conducted sequential analyses examining potential underlying factors responsible for older adults’ distrust in online health information. We first looked at the bivariate relationships between all independent variables and trust. We then conducted three multivariate logistic regressions, adding sequentially groups of variables that we hypothesized might influence the relationship between age and trust. In Model 1, we included sociodemographic characteristics (sex, race, and education), as well as the respondent’s self-reported health status and functional limitations. In Model 2, we added years of Internet experience and frequency of technical difficulties with the computer or Internet. In Model 3, we added other hypothesized reasons for distrust: feelings of frustration or confusion toward online health information, and lack of awareness about the source providing the online health information. We also examined our full model after adjusting for individuals’ trust in all non-Internet sources of health information.

Regression diagnostic procedures yielded no evidence of multicolinearity in any of the regression models (mean variance inflation factor = 1.21). Rates of item-level missing data were less than 5% for all independent variables used in analyses. Survey weights were used to adjust for the sampling design of the study. We performed all analyses using Stata version 11.0 (StataCorp, College Station, TX). All data were deidentified prior to acquisition of the dataset from the Kaiser Family Foundation.

## Results

The mean (SD) age of the overall study population was 63.7 (10.6) years. There were 823 (56.8%) respondents who reported using the Internet, and the mean (SD) age of this subgroup was significantly younger than the subgroup of individuals who had never used the Internet, at 59.3 (8.1) versus 69.4 (10.7), *P* < .001. Among the Internet users, 745/823 (90.6%) reported having a computer at home, 700 (85.2%) reported having Internet access at home, and 404 (49.6%) reported having 5 or more years of Internet experience. There were 411/811 respondents (50.2% of Internet users) who reported using the Internet daily. Additional characteristics of the study population are described in Table 1.

**Table 1:** Characteristics of the study population

			Total population (N = 1450)			Online population (n = 823)		
			N	n	%	N	n	%
**Sociodemographics and health status**								
	Age, mean (SD)		63.7 (10.6)			59.3 (8.1)		
		≥65 years	1382	583	42.2	782	190	24.3
	Sex, female		1450	915	63.1	823	489	59.4
	Race, white		1409	1213	86.1	803	717	89.3
	Education		1426			818		
		Less than high school		171	12.0		27	3.3
		High school graduate		520	36.5		216	26.4
		Some college		337	23.6		244	29.8
		College graduate		398	27.9		331	40.5
	Employed		1431	620	43.3	815	480	58.9
	Household income		1039			633		
		<$30,000		398	38.3		124	19.6
		$30,000-100,000		506	48.7		381	60.2
		>$100,000		135	13.0		128	20.2
	Fair or poor health status		1443	303	21.0	819	108	13.2
	Functional limitations due to disability or chronic disease		1441	306	21.2	822	138	16.8
	Primary caregiver for household member		1437	231	16.1	822	121	14.7
**Computer and Internet Use**								
	Computer at home		1449	901	62.1	822	745	90.6
	Internet access at home		1449	785	54.2	822	700	85.2
	High-speed Internet access at home		1449	314	21.7	822	293	35.6
	Internet experience >5 years		1449	404	27.9	814	404	49.6
	Frequency of Internet use		1445			818		
		Never		627	43.2		—	—
		Less than weekly		111	7.7		111	13.6
		Weekly		296	20.5		296	36.2
		Daily		411	28.4		411	50.2
	Frequency of Internet use for health information		1445			823		
		Never		822	56.9		195	23.7
		Less than monthly		389	26.9		389	47.3
		Once or twice per month		158	10.9		158	19.2
		At least weekly		76	5.3		76	9.2
	Trust in Internet as a source of health information		1291			802		
		Not at all		480	37.2		124	15.5
		Not too much		125	9.7		89	11.1
		Somewhat		478	37.0		404	50.4
		A lot		208	16.1		185	23.1

There were 628 respondents who reported having used the Internet as a health resource (76.3% of Internet users), and more than a third of them did so at least once a month (Table 1). Among Internet users, age was not a predictor of a person having used the Internet as a health resource (Table 2), and this remained true when we examined age as a continuous variable (adjusted OR 0.99, *P* = .64). Older adults were less likely than those under age 65 years to report that the Internet had helped them care for their health (OR 0.54, *P* = .02), but this effect was diminished after adjusting for trust, years of Internet experience, and other covariates (adjusted OR 0.65, *P* = .11). Trust, however, was significantly associated with using the Internet as a health resource (adjusted OR 4.84, *P* < .001) and with finding the Internet useful as a health resource (adjusted OR 3.74, *P* < .001).

**Table 2:** Characteristics associated with a person using the Internet for health information and finding the Internet useful as a health resource

			Ever used the Internet for health information, N = 823 (Internet users)				Finds the Internet useful as a health resource, N = 628 (online health information users)			
			Unadj OR	*P*-value	Adj OR	*P*-value	Unadj OR	*P*-value	Adj OR	*P*-value
**Main variables of interest**										
	Age ≥65 years^a^		0.78	.27	1.19	.48	0.54	.02	0.65	.11
	Trust (somewhat/a lot) in Internet as source of health information^b^		5.87	<.001	4.84	<.001	3.75	<.001	3.74	<.001
**Other covariates**										
	Female		1.27	.22	1.48	.10	1.41	.08	1.19	.44
	Nonwhite		1.29	.45	1.31	.50	1.53	.18	2.02	.07
	Education^b^									
		Some post-high school education	1.48	.12	1.34	.33	1.15	.59	1.15	.64
		College graduate	1.84	.01	1.43	.23	1.04	.87	0.83	.54
	Fair/poor health status^b^		0.92	.78	0.95	.90	1.18	.60	1.03	.93
	Functional limitations due to disability or chronic disease		1.28	.39	1.86	.17	0.89	.66	0.94	.85
	Internet experience >5 years		1.91	.002	1.77	.035	1.30	.19	1.36	.21
	Technical difficulties with computer/Internet		0.91	.52	0.98	.91	0.95	.68	0.97	.85

^a^ Age is presented as a dichotomous variable for clarity. When age is analyzed as a continuous variable, the relationship with ever using the Internet for health information remains nonsignificant, but the relationship with finding the Internet useful as a health resource is significant (adjusted OR 0.97, *P* = .03).

^b^ Comparison group is “not at all/not too much” for trust in online health information, “high school or less” for education, and “excellent/very good/good” for health status.

Trust was significantly associated with the performance of a number of online health-related activities, even after adjusting for sociodemographic and health characteristics. Individuals who reported trusting the Internet “somewhat” or “a lot” for health information were significantly more likely to report that they had searched for information about a specific health condition (adjusted OR 4.43, *P* < .001), and that they had used the Internet to obtain information about topics ranging from their health care provider (adjusted OR 2.24, *P* = .007), to health policy news (adjusted OR 3.37, *P* = .007), to prescription drug prices (adjusted OR 4.93, *P* < .001). They were also significantly more likely to purchase prescription drugs and nutritional supplements online (adjusted OR 2.61, *P* = .03 and adjusted OR 3.43, *P* = .002, respectively). In addition, among the 628 respondents who had any history of using the Internet for health information, those with higher levels of trust were more likely to change their behavior because of the online information (adjusted OR 2.15, *P* = .03), and they were more likely to talk with a health care provider about the online information (adjusted OR 2.54, *P* = .002).

Finally, in a series of multivariate logistic regression models in which we sequentially added variables of interest, we examined the relationship between age and trust in the Internet as a source of health information (Table 3). In bivariate analyses, Internet users 65 years of age and older were significantly less likely than those younger than 65 to report trusting the Internet for health information (OR 0.63, *P* = .04). This relationship persisted after adjusting for other sociodemographic and health characteristics (Model 1), and was only slightly attenuated (adjusted OR 0.69, *P* = .13) after adjusting for Internet experience and technical difficulties with computers and the Internet (Model 2). The age effect disappeared entirely (adjusted OR 0.96, *P* = .91), however, after adjusting for other hypothesized contributors to distrust (Model 3), such as finding the Internet confusing because it provides “too much information” (adjusted OR 0.47, *P* = .03), and not routinely identifying the provider of online health information (adjusted OR 0.53, *P* = .04). These relationships were virtually unchanged when we adjusted for overall trust levels in non-Internet sources of health information.

**Table 3:** Characteristics associated with trust in the Internet as a source of health information among adults ≥50 years of age who use the Internet (n = 823)

			Bivariate relationships		Model 1: age, sex, race, education, and health and function		Model 2: Model 1 + Internet experience and technical difficulties		Model 3: Model 2 + other hypothesized reasons for distrust	
			Unadj OR	*P*-value	Adj OR	*P*-value	Adj OR	*P*-value	Adj OR	*P*-value
Age ≥65 years ^a^			0.63	.04	0.63	.04	0.69	.13	0.96	.91
Female			1.48	.04	1.67	.01	1.79	.01	1.46	.21
Education^b^										
		Some post-high school education	1.45	.13	1.31	.29	0.91	.73	0.68	.30
		College graduate	2.47	<.001	2.53	<.001	1.81	.04	0.98	.96
Nonwhite			1.21	.55	1.13	.73	1.13	.75	0.90	.84
Fair/poor health status^b^			0.80	.43	0.90	.74	1.42	.37	1.79	.32
Functional limitations due to disability or chronic disease			0.70	.17	0.89	.71	1.03	.93	1.09	.85
Internet experience >5 years			1.62	.02	—		1.78	.02	1.25	.50
Technical difficulties with computer/Internet			0.89	.41	—		1.03	.84	0.92	.69
Negative feelings toward online health information										
	Frustrating: hard to find what is needed		0.49	.009					0.99	.99
	Confusing: too much information		0.53	.02	—		—		0.47	.03
Lack of awareness (never/hardly ever) of source providing health information found online^b^			0.42	.002	—		—		0.53	.04

^a^ Age is presented as a dichotomous variable for clarity. When age is analyzed as a continuous variable, the adjusted ORs and *P*-values for Models 1, 2, and 3 are as follows: Model 1 = 0.96 (*P* = .002), Model 2 = 0.96 (*P* = .006), Model 3 = 0.99 (*P* = .77). Adjusted ORs and *P*-values for other covariates in the models are essentially unchanged when age is analyzed as a continuous variable.

^b^ Comparison group is “high school or less” for education, “excellent/very good/good” for health status, and “sometimes/mostly/always” for awareness of online health information source.

## Discussion

In this nationally representative survey of adults aged 50 years and older, we found that individuals who reported trusting the Internet as an information source were significantly more likely to report that the Internet had helped them care for their health, and were also more likely to use the Internet for a number of important health-related activities, including searching for information about a specific health condition, comparing prescription drug prices and purchasing medications, obtaining information about a health care provider, and talking with their provider about information found online.

While the relationship between trust and Internet use appears intuitive, there are many circumstances in which distrust in online health information is appropriate [17]. The Internet lacks an effective quality control mechanism, and this, combined with the ease of replicating online material, leads to the spread of false information [20]. Older adults, many of whom use the Internet for a relatively limited number of functions and are unfamiliar with a metric for trustworthiness, are likely to have low levels of autonomy and may not have the tools that are required to assess the credibility of online health information. Our findings suggest that older adults’ distrust may be a significant barrier to their optimal usage of the Internet for their health. This is of concern, given that the Internet offers an efficient means to obtain information and conduct important health-related activities, and many websites today provide reliable, up-to-date, and sometimes tailored health information. Such a resource could be especially valuable for someone who is homebound due to multiple health problems or because of their caregiving obligations.

As the Internet’s capabilities as a health resource expand, it is important that older individuals be provided with tools and knowledge to assess the credibility of online health information [15]. This is especially critical given that dubious information regarding medical issues can result in physical or mental harm [8]. The results of our study highlight several potential targets for improving older adults’ trust in the Internet as a health resource. While adults aged 65 years and older were significantly less likely to trust the Internet for health information, this association disappeared after accounting for two significant factors: one was confusion due to overwhelming amounts of information, and the other was lack of awareness about the source providing health information found online (a key step to assessing the credibility of a website). These issues could be addressed through websites that incorporate senior-friendly design elements (eg, an uncluttered layout with a large font size and comfortably sized buttons and links) [7,21] and through the promotion of websites that are clearly associated with trustworthy institutions (ie, via credibility cues like images and logos) [22]. Clarifying the source and credibility of information may be especially important for individuals with lower levels of autonomy who tend to gravitate toward traditional intermediaries for health information [8].

An example of a site that embraces these concepts is the NIH Senior Health website, which presents information from government agencies such as the National Cancer Institute and the National Institute on Aging about a multitude of health conditions. The website is tailored to meet the needs of an older, less-experienced Internet user, with a simple design, and options to increase the text size, enhance contrast, and hear the text read aloud [23]. The growth of health portals may provide other trustworthy sources of information. Portals such as those developed for patients of Kaiser Permanente [24], Group Health Cooperative [25], and the Veterans Affairs Healthcare System [26] direct users to reliable information and expertise that is often personally tailored to an individual’s specific health needs [10]. A survey of Kaiser Permanente patients found that 87% of respondents over the age of 65 years were satisfied with Kaiser’s My Health Manager [27]. The role of such portals for older individuals who distrust or avoid using the Internet for their health, however, remains to be seen.

While the data for our study were derived from a nationally representative sample of adults aged 50 years and older, the structure of the survey introduced some limitations. Our reliance on an existing dataset necessitated the use of available measures, including measures that were created de novo for the survey and have not been externally validated. There may be unmeasured characteristics that influence a person’s trust in online health information but were not assessed in this survey, such as general trustfulness and specific health issues and information needs. In addition, the data in this study were cross-sectional, and as such we cannot make any conclusions about causality or mediation in our analyses. Biases may have resulted from nonresponse and from self-reported data, with common method variance potentially explaining some degree of the high level of internal consistency we saw in certain constructs. Finally, our use of 2004 data is also a limitation, given demographic changes in Internet usage and the rise of new website features, such as the option of communicating with health care providers online, and the growth of well-informed online patient communities. Nevertheless, this survey covered unique territory, and there is reason to believe that the association between trust and information-seeking behavior could transcend the developments since the survey was conducted.

In conclusion, in this nationally representative sample of older adults, we found that trust in online health information is significantly linked to use of the Internet for a wide range of health-related purposes. While the association between distrust and diminished use of the Internet is not surprising, the strength and consistency of this relationship suggests that building trust, in part through the development and promotion of simple and credible websites and health portals, is likely to be a crucial step in improving the accessibility and utility of online health resources for older adults. Future research should focus on identifying the specific design features, content, and functions that will optimize the value of such resources.

## Abbreviations

PSRA: Princeton Survey Research Associates
